# Pulmonary Resection after Radiosurgery and Neoadjuvant Immunochemotherapy for NSCLC Patients with Synchronous Brain Metastasis—A Case Series of Three Patients

**DOI:** 10.3390/curroncol29040181

**Published:** 2022-03-23

**Authors:** Agnes Koch, Stefan Sponholz, Stephan Trainer, Jan Stratmann, Martin Sebastian, Maximilian Rauch, Robert Wolff, Joachim P. Steinbach, Michael W. Ronellenfitsch, Hans Urban

**Affiliations:** 1 Department of Thoracic Surgery, Agaplesion Markuskrankenhaus, 60431 Frankfurt am Main, Germany; stefan.sponholz@fdk.info (S.S.); stephan.trainer@fdk.info (S.T.); 2 Hematology/Oncology, Department of Medicine, University Hospital Frankfurt, Goethe University, 60590 Frankfurt am Main, Germany; jan.stratmann@kgu.de (J.S.); martin.sebastian@kgu.de (M.S.); 3 University Cancer Center Frankfurt (UCT), University Hospital Frankfurt, Goethe University, 60590 Frankfurt am Main, Germany; maximilian.rauch@kgu.de (M.R.); joachim.steinbach@med.uni-frankfurt.de (J.P.S.); michael.ronellenfitsch@kgu.de (M.W.R.); 4 Frankfurt Cancer Institute (FCI), 60590 Frankfurt am Main, Germany; 5 German Cancer Consortium (DKTK), Partner Site Frankfurt am Main, German Cancer Research Center (DKFZ), Stiftung des Öffentlichen Rechts, 69120 Heidelberg, Germany; 6 Institute of Neuroradiology, University Hospital Frankfurt, Goethe University, 60528 Frankfurt am Main, Germany; 7 Saphir Radiosurgery Center, 60528 Frankfurt am Main, Germany; r.wolff@gkfrankfurt.de; 8 Dr. Senckenberg Institute of Neurooncology, University Hospital Frankfurt, Goethe University, 60528 Frankfurt am Main, Germany

**Keywords:** neoadjuvant immunochemotherapy, NSCLC, brain metastasis, immune checkpoint inhibitors, pembrolizumab, stereotactic radiosurgery

## Abstract

**Simple Summary:**

In this short communication, we present three cases of patients with symptomatic, synchronous brain metastases of otherwise locally limited non-small cell lung cancer. The patients received local ablative treatment of the brain metastases followed by neoadjuvant immunochemotherapy with pemetrexed, cisplatin, and pembrolizumab, and resection of the pulmonary lesion with curative intent. With two of the patients still alive and maintaining a good quality of life with a progression-free survival and overall survival of 28 and 35 months, respectively, this case series illustrates the potential of novel combinatorial treatment approaches.

**Abstract:**

Brain metastases are a common finding upon initial diagnosis of otherwise locally limited non-small cell lung cancer. We present a retrospective case series describing three cases of patients with symptomatic, synchronous brain metastases and resectable lung tumors. The patients received local ablative treatment of the brain metastases followed by neoadjuvant immunochemotherapy with pemetrexed, cisplatin, and pembrolizumab. Afterwards, resection of the pulmonary lesion with curative intent was performed. One patient showed progressive disease 12 months after initial diagnosis, and passed away 31 months after initial diagnosis. Two of the patients are still alive and maintain a good quality of life with a progression-free survival and overall survival of 28 and 35 months, respectively, illustrating the potential of novel combinatorial treatment approaches.

## 1. Introduction

Lung cancer remains the leading cause of cancer deaths worldwide [1]. Non-small cell lung cancer (NSCLC) accounts for 85–90% of lung cancers [2], and is often discovered in later stages of the disease. In patients with stage IVA oligometastatic disease and resectable lung tumors, a curative approach may be feasible [3].

**Figure 1:**
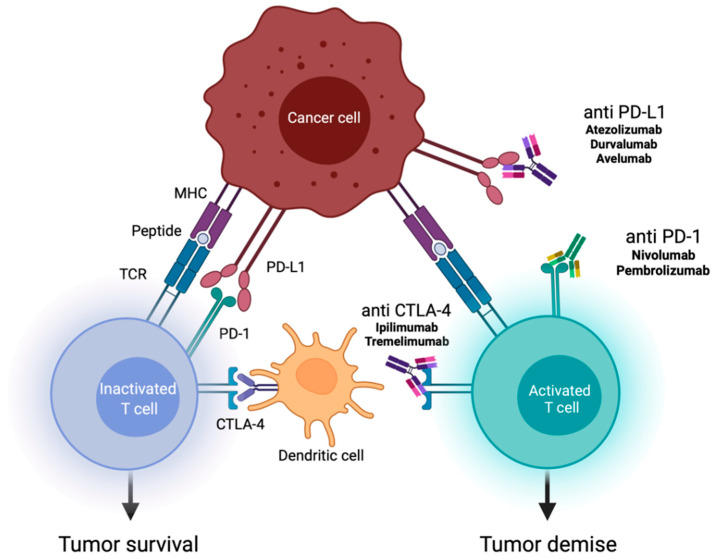
Mechanisms of action of immune checkpoint inhibitors (ICIs). This figure shows the role of ICIs in activating the immune system against tumor cells. The various immune checkpoint proteins are expressed by T cells on their surface, and interact with their ligands on antigen-presenting cells (e.g., dendritic cells). ICIs (antibodies against programmed death 1 (PD-1)/programmed death ligand 1 (PD-L1), and against cytotoxic T-lymphocyte-associated antigen 4 (CTLA-4)) stop the interaction of receptors and ligands, and promote T cell-mediated immune response [8,9].

Immune checkpoint inhibitors (ICIs) are one of the most important clinical advances for a wide range of malignancies, especially for lung cancer—a disease that frequently metastasizes to the brain. The mode of action of ICIs involves activation of T-cell effector functions via the inhibition of T-cell immune checkpoints (Figure 1). Pembrolizumab is an antibody ICI that targets the programmed cell death protein 1 (PD-1), and is approved for combination therapy with pemetrexed and platin-based chemotherapy as a first line treatment for non-squamous NSCLC without epidermal growth factor receptor (EGFR) mutation or anaplastic lymphoma kinase (ALK) translocation (Figure 1). In this patient group, addition of pembrolizumab significantly improves overall survival (OS) and progression-free survival (PFS) compared to treatment with only pemetrexed and platinum-based chemotherapy. The KEYNOTE-189 trial showed an OS of 22.0 versus 10.6 months, but patients with symptomatic brain metastases were excluded [4].

Brain metastases are a common complication of NSCLC. About 20% of NSCLC patients present with synchronous brain metastases upon initial diagnosis [5]. The treatment of brain metastases has made great progress in recent years, and a combination of checkpoint inhibitors and radiotherapy [6] has shown promising results. Because of the increasing chance for local control of brain metastases, e.g., via stereotactic radiosurgery (SRS) or neurosurgery, treatment of the primary tumor is coming back into focus. The current ASCO guideline recommends local therapeutic measures to control symptomatic brain metastasis, e.g., radiosurgery and/or radiation therapy and/or surgery [7]. However, the role of complete resection of the thoracic tumor in the presence of locally treatable brain metastases is an important aspect. In the following, we present three cases of such a treatment approach.

## 2. Materials and Methods

The patients in this retrospective case series were all treated by the thoracic surgery department of Agaplesion Markus Hospital (Frankfurt, Germany) between June 2019 and February 2020. Since the establishment of the department in 2017, they were the only patients with oligometastatic NSCLC adenocarcinoma exclusively to the brain who received neoadjuvant immunochemotherapy prior to surgical resection of the lung tumor.

**Figure 2:**
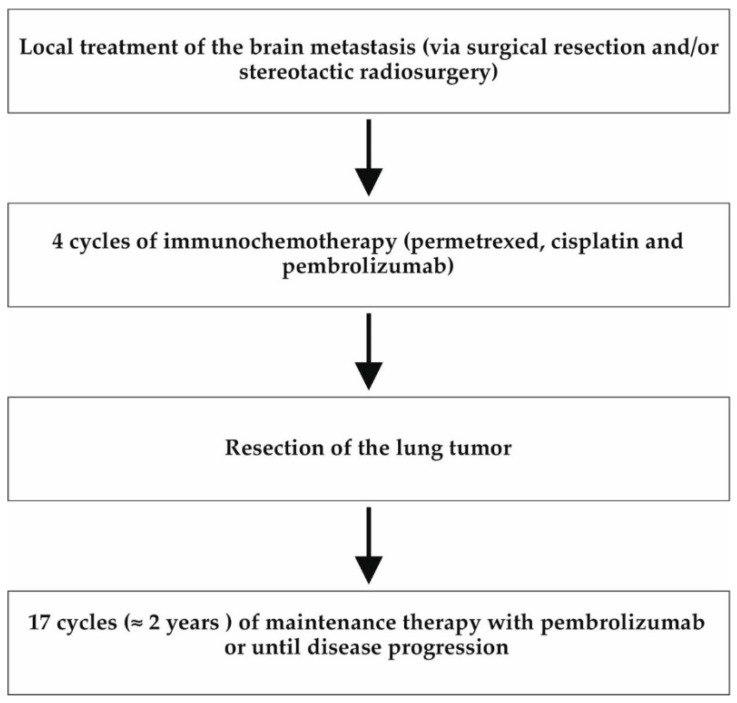
Flow chart of the patients’ treatment plan. All three patients were initially treated according to the following treatment algorithm in line with the ASCO guidelines [7]. At the beginning, local therapy of the brain metastasis was performed by means of neurosurgery or stereotactic radiosurgery. Afterwards, the patients received four cycles of immunochemotherapy followed by resection of the primary lung tumor. This was followed by maintenance immunotherapy for 2 years (or until disease progression).

All patients were discussed in a multidisciplinary tumor conference including an oncologist, a thoracic surgeon, a radiation therapist, a radiologist, and a psycho-oncologist. All patients were also discussed in a specialized brain tumor conference which included a neurooncologist, a neurosurgeon, a radiation therapist, a neuroradiologist, a neuropathologist, and a radiosurgery specialist. The patients were all in an oncological treatment plan of oligometastatic lung cancer, including local therapy of the brain metastasis, immunochemotherapy, and resection of the primary lung cancer. The patients were not part of any further study protocol. After four cycles of immunochemotherapy, the tumor response was evaluated by a chest computed tomography (CT) scan or positron emission tomography (PET) scan. Progression of the tumor after immunochemotherapy would have been a contraindication for lung tumor resection. Furthermore, to preclude new metastases, a complete imaging of the lung, abdomen, brain, and bones was required before the operation. Patients received an echocardiogram, a cardiac stress test, and a pulmonary function test in order to evaluate functional operability, as well as a preoperative bronchoscopy. There were no contraindications to the lung cancer operation in our patients. After resection of the lung tumor was performed, the patients received maintenance immunotherapy with pembrolizumab, which was administered for two years (corresponding to 17 cycles) or until progression of the disease occurred (Figure 2).

The patients or their legal guardians gave their consent to the publication of these data. We thank the patients and their families for their support.

## 3. Results

### 3.1. Case 1

**Figure 3:**
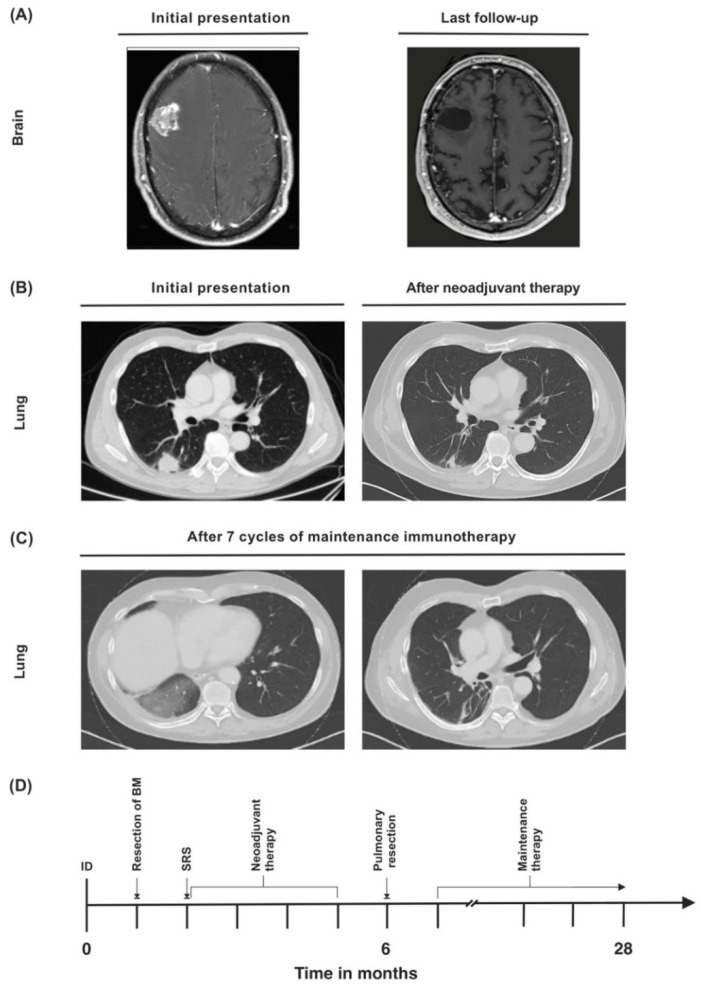
(**A**) Synchronous cerebral metastasis at baseline and upon last follow-up. (**B**) Primary lung tumor at initial diagnosis and after neoadjuvant therapy. (**C**) Lung infiltrations in the right lung that were attributed to immunotherapy-associated pneumonitis were detected during maintenance therapy. (**D**) Timeline of the patient’s treatment course. ID = initial diagnosis.

A 56-year-old Caucasian male presented in August 2019 with mnestic deficits, mild motoric aphasia (word-finding difficulty), and progressive numbness of the left side of the face. These symptoms had been present for several weeks (Eastern Cooperative Oncology Group (ECOG) 1, Karnofsky performance score (KPS) 90%). The patient history included former nicotine abuse (cumulative 120 pack years (PYs)), multiple herniated discs, Lyme disease (stage 1), and scleroderma. The patient did not take any regular medications at presentation; a past drug history included methotrexate to treat scleroderma 2 years ago. A cranial magnetic resonance imaging (MRI) scan showed a mass (2.9 × 2.7 cm) in the right frontal lobe with pronounced perifocal edema and contrast agent enhancement (Figure 3A).

A chest CT scan revealed a 3 × 3 cm sized tumor in the right lower pulmonary lobe in the absence of lymph node enlargement (Figure 3B). CT-guided fine needle biopsy yielded a moderately differentiated thyroid transcription factor 1-(TTF1)-positive NSCLC adenocarcinoma of the lung (Table 1). A bronchoscopy with endobronchial ultrasound was performed, but since there were no enlarged lymph nodes, no endobronchial biopsy was taken.

In addition, the patient received a functional cranial MRI, indicating the speech center to be located on the right side of the patient’s brain. Despite the proximity to the speech center, a craniotomy with excision of the suspected metastasis was performed. The patient received postoperative radiation of the tumor bed via SRS. Clinical tumor stage was concluded to be cT2 cN0 pM1b (brain (BRA)), Union for International Cancer Control (UICC) stage IVA.

A subsequent immunochemotherapy of four cycles of cisplatin, pemetrexed, and pembrolizumab was performed. Due to edema after local ablation of the brain metastasis, an accompanying therapy with dexamethasone was administered during systemic therapy.

In the concept of a multimodal therapeutic regime after completion of neoadjuvant therapy, in February 2020, a posterolateral thoracotomy with extended resection of the right lower lobe (including partial resection of the right atrium; resection of subsegment 1; as well as a systematic mediastinal, hilar, and interlobar lymph node dissection) was performed. Interlobar preparation was challenging due to fibrotic alteration of the tissue after neoadjuvant therapy. Because of postoperative hemoptysis, the patient required three bronchoscopies to remove coagulated blood from the bronchial system. Also, postoperative tachyarrhythmia occurred, which subsisted under treatment with metoprolol. Because of a CHA_2_DS_2_-VASc (congestive heart failure, hypertension, age ≥75 years (double weight), diabetes mellitus, stroke (double weight), vascular disease, age 65–74 years, female sex) score of 0 and postoperative hemoptysis, therapeutic anticoagulation was not administered. The patient was discharged in good general condition on the 14th postoperative day. Pathology analysis showed a complete regression of the tumor with a postoperative stage of ypT0 ypN0 (0/42), L0, V0, R0, Gx, pM1b (BRA), UICC stage IVA.

After recovery from surgery, the patient was able to return to the workplace. Now free of any tumor manifestation, he was put on an adjuvant course of pembrolizumab that he is planned to receive for two years.

Regular follow-up examinations showed postoperative scarring without any evidence of recurrent disease. A CT scan 12 months after initial diagnosis detected infiltrations in the right lung which could be interpreted as immunotherapy-associated pneumonitis (Figure 3C). However, the patient did not display any symptoms, such as cough or dyspnea, and immunotherapy was continued. Twenty-eight months after initial diagnosis, the patient is still alive in good general condition, and shows no evidence of recurrent or metastatic disease (Figure 3D). He is still receiving pembrolizumab, which he is tolerating well.

### 3.2. Case 2

**Figure 4:**
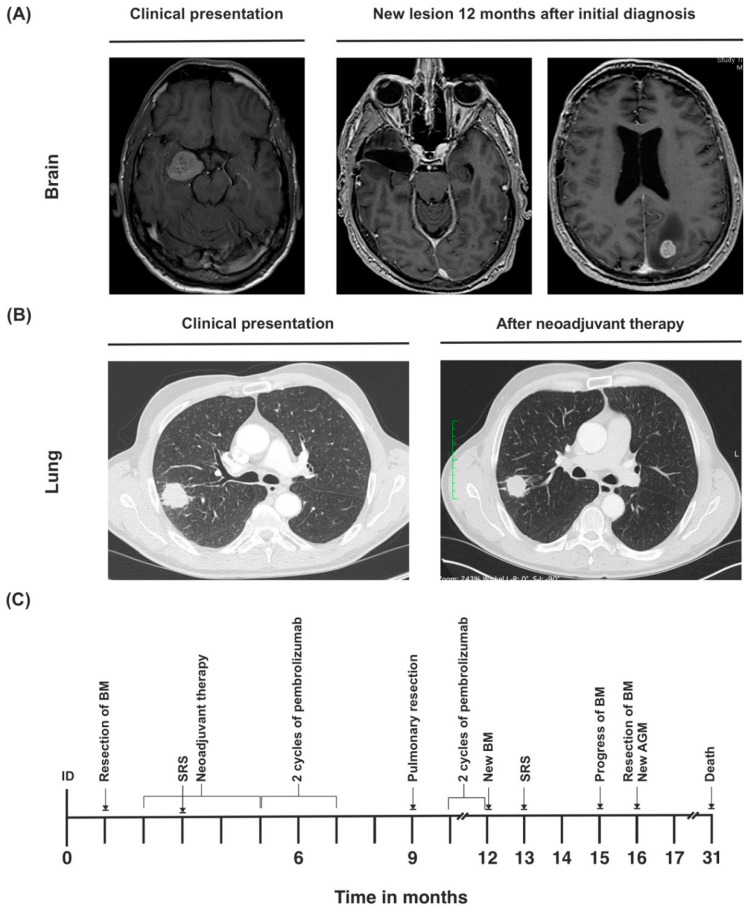
(**A**) Cranial MRI of synchronous metastasis upon initial diagnosis, and second metastasis that developed during the course of disease. (**B**) Primary tumor at initial diagnosis and after neoadjuvant therapy. (**C**) Timeline of the patient’s treatment course. ID = initial diagnosis.

A 53-year-old Caucasian male initially presented with double vision due to an oculomotor palsy of the right eye (ECOG 1, KPS 90%) in March 2019. Patient history was unremarkable except for former nicotine abuse (cumulative 60 PYs). The cranial MRI showed a 3 × 2.5 cm sized mass medial to the right temporal lobe with slight edema (Figure 4A). An extirpation was performed via frontotemporal access, revealing likely metastasis of a poorly differentiated, TTF-1-negative adenocarcinoma (Table 1).

Postoperatively, the patient still suffered from oculomotor nerve deficits with ptosis, mydriasis, and abduction of the right eyeball. He also had mild alteration of the frontal lobe function. The tumor markers were within normal range (carcinoembryogenic antigen (CEA) 6.6 ng/mL, neuron specific enolase (NSE) 15.1 mg/L, and prostate specific antigen (PSA) 0.48 ng/mL). Chest CT showed a 3.7 × 2.7 cm sized tumor in the right upper pulmonary lobe with pleural adhesion and a right hilar lymph node enlargement (Figure 4B). A PET scan was performed to further clarify these findings, which showed a strongly increased fluorodeoxyglucose (FDG) uptake of the suspected primary lesion in the right upper lobe. Elevated FDG uptake of the hilar and mediastinal lymph nodes was also observed, though not as high as in the primary lesion. It was concluded that the patient suffered from oligometastatic lung cancer that had caused a single brain metastasis (tumor stage cT2b cN2 pM1b (BRA), UICC stage IVA).

The tumor bed of the cerebral resection cavity was treated with postoperative radiation via Cyber Knife. A combined immunochemotherapy consisting of four cycles of cisplatin, pemetrexed, and pembrolizumab was performed from May until August 2019. Afterwards, the patient was put on a maintenance therapy with pembrolizumab.

During the treatment, the patient developed structural epilepsy, which was successfully treated with levetiracetam.

Restaging was performed before the planned resection of the primary lesion. Cranial MRI showed no evidence of recurrent metastasis. PET scan showed no progression of the primary lesion. There was still a moderately elevated FDG uptake of the hilar and mediastinal lymph nodes. There was neither a change in size nor an increase in FDG avidity of the left adrenal gland in comparison to the earlier imaging. A bronchoscopy with endobronchial ultrasound and transbronchial needle aspiration biopsy was performed to further investigate the hilar and mediastinal lymph nodes. The left lower paratracheal node (station 4L), infracarinal lymph node (station 7), left hilar lymph node (station 10L), and right hilar lymph node (station 10R) were biopsied. Histopathological examination showed silicoanthracosis without any evidence of malignancy.

In December 2019, the primary lesion was resected via posterolateral thoracotomy with extrapleural extended upper lobectomy; wedge resections of segment 1, segment 5, and segment 6; and a systematic hilar, mediastinal, and interlobar lymph node dissection. There was a marked increase in vascularization and adhesions due to previous immunochemotherapy, especially in the upper mediastinum. During the course of recovery, the patient developed a chest wall seroma that required temporary insertion of a Redon drain.

The histopathological report showed a fully resected, solid NSCLC adenocarcinoma. Postoperative tumor stage was ypT2b (4.5 cm) ypN0 (0/22), L0, V0, Pn0, R0, Gx, pM1b (BRA), UICC stage IVA. The patient was discharged on the 19th postoperative day.

After the operation, the patient continued maintenance therapy with pembrolizumab. In March 2020, 12 months after initial diagnosis, cMRI showed a new 4 mm-sized metastatic lesion in the left occipital lobe (Figure 4A). Due to progressive disease, pembrolizumab was discontinued. The metachronous brain metastasis was treated with radiosurgery via Cyber Knife.

In June 2020, the patient developed generalized epileptic seizures. Cranial MRI showed that although the metastasis had not significantly progressed in size, an extensive edema had emerged around it. In July 2020, CT scan of the abdomen revealed a progression in size to the left adrenal gland, indicating metastatic disease. Also, cranial MRI showed a new growth at the border of the prior radiation site (Figure 4A). Due to uncontrollable epileptic seizures, surgical resection of the brain metastasis was performed in August 2020. Pathology report showed a fully resected metastasis of the known poorly differentiated adenocarcinoma with a significant change in programmed death-ligand 1 (PD-L1) status (synchronous brain metastasis with moderate PD-L1 expression, metachronous brain metastasis with low PD-L1 expression). The patient continued to suffer from generalized epileptic seizures, and showed signs of organic brain psychosyndrome, endangering himself and others.

Due to poor prognosis, therapy regimen was switched to best supportive care. The patient passed away 31 months after initial diagnosis (Figure 4C).

### 3.3. Case 3

**Figure 5:**
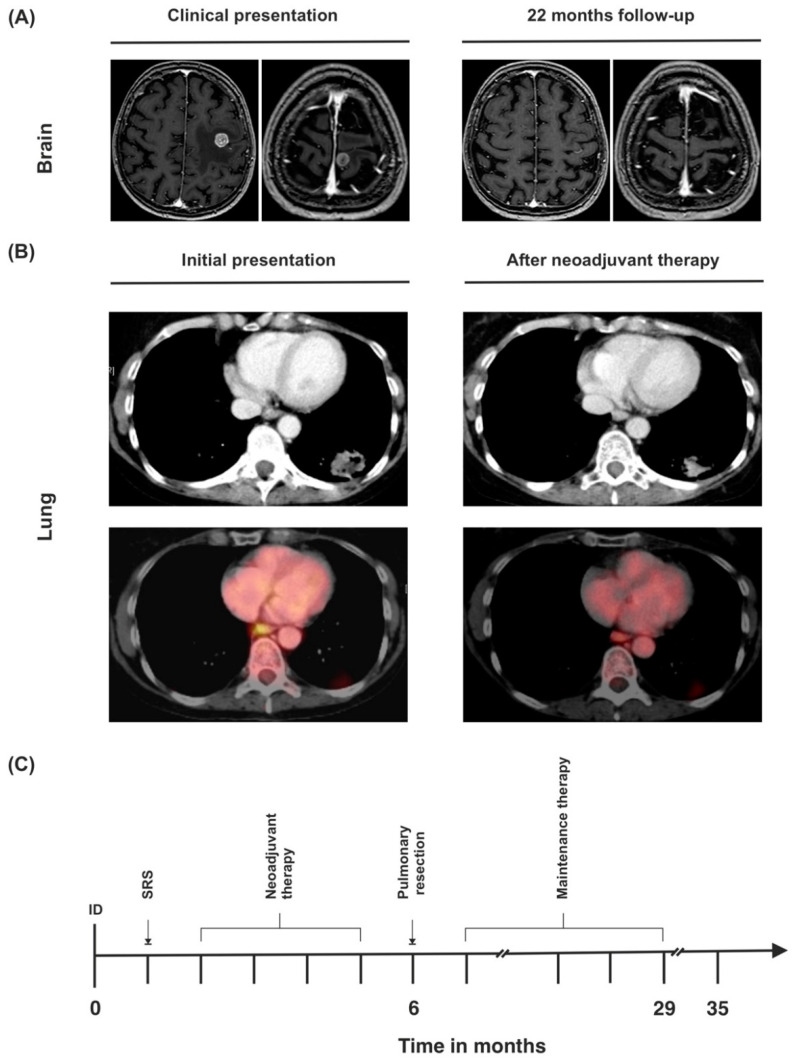
(**A**) MRI of the brain upon initial diagnosis and last follow-up. (**B**) CT and PET-CT findings of the primary tumor at the time of initial diagnosis and after neoadjuvant therapy. Left hilar lymph nodes (SUV_max_ 3.3) before neoadjuvant therapy and after neoadjuvant therapy (SUV_max_ not detectable). (**C**) Timeline of the patient’s treatment course. ID = initial diagnosis.

In January 2019, a 58-year-old Caucasian female presented in the emergency room with impairment of fine motor skills, dizziness, apraxia, and weakness in the right arm (abnormal posturing—pronation and sinking), which had been present for 4 days (ECOG 1, KPS 90%). Preexisting conditions included chronic obstructive pulmonary disease (COPD), emphysema of the lung, and continued nicotine abuse (cumulative 60 PYs). Two cerebral lesions were found to be the cause of the symptoms, the larger one (11 × 10 mm) being located in the left pre-central cortex, and accompanied by pronounced edema. The smaller lesion in the gyrus postcentralis (9 × 6.5 mm) showed only slight edema (Figure 5A).

Dexamethasone was administered for 3 weeks, under which the symptoms subsided. Chest CT showed a 3.3 × 2.1 cm sized mass in the left lower pulmonary lobe with possible pleural infiltration (Figure 5B). CT-guided fine needle biopsy revealed a TTF-1 positive NSCLC adenocarcinoma (Table 1). Further staging via PET CT showed a strongly elevated FDG uptake of the primary lesion (standardized uptake value (SUV_max_) 15.5, Figure 5B). There was also an elevated FDG uptake in the left hilar lymph nodes, left paraesophageal, and left parailiacal lymph nodes (SUV_max_ 3.3). A slightly enlarged (1.6 cm) left adrenal gland was observed on abdominal CT scan without any FDG avidity. Clinical tumor stage was concluded to be cT2 cN3 cM1c (BRA), UICC stage IVB.

Because of oligometastatic disease and the good general condition of the patient, local ablation of both brain metastases via SRS and a systemic immunochemotherapy consisting of four cycles of cisplatin, pemetrexed, and pembrolizumab was carried out. Restaging via PET scan showed partial necrosis of the primary lesion, a slight decrease in size to 3 × 2.1 cm, and a reduction of SUV_max_ from 15.5 to 9.0. There was no elevated FDG uptake in any of the lymph nodes that had been PET-positive on the first scan. However, the left hilar lymph nodes showed a progression from 0.5 cm to 0.8 cm on chest CT. Lymph node status was therefore difficult to assess (Figure 5B).

The primary lesion in the left lower lobe, as well as the potentially affected lymph nodes, were deemed to be fully resectable. The patient underwent a left posterolateral thoracotomy; extended left lower lobe resection; wedge resection in segment 1; and a systematic dissection of the hilar, mediastinal, and interlobar lymph nodes. There was extensive scarring and fibrotic tissue after prior immunochemotherapy, especially around the pulmonary artery, the interlobar fissure, and the mediastinal and hilar lymph nodes. Histopathological examination showed a fully resected, TTF-1-positive NSCLC adenocarcinoma with postoperative tumor stage ypT2a (3.5 cm) ypN3 (5/23), L1, V1, Pn0, R0, cM1c (BRA), UICC stage IVB. The N3 lymph node metastasis (right hilar lymph node, position 10R) showed growth beyond the lymph node’s margin, but had been completely resected en bloc with the surrounding fatty tissue.

After an uneventful recovery, the patient was discharged on the 10th postoperative day, and resumed maintenance treatment with pembrolizumab, which she received for two years without any problems. Thirty-five months after initial diagnosis, chest and abdominal CT scan and cranial MRI showed no sign of local recurrence or metastatic disease (Figure 5C).

## 4. Discussion

The treatment of metastatic NSCLC is challenging because it requires multimodal therapy with seamless communication between all involved clinical disciplines. For stage IVA disease with brain metastases, local treatment of single brain metastases and resection of the primary lesion can be considered in highly selected patients [3]. Although treatment with immune checkpoint inhibitors (ICIs) is common in the clinical routine in patients with brain metastases, patients with synchronous, symptomatic brain metastases have so far been excluded from pivotal trials.

Even in metastasized disease, surgical resection of the primary tumor and/or metastasis results in a prolonged survival [10]. Studies from the pre-checkpoint era show a limited median OS of 12.1 months [11] in patients with synchronous brain metastasis who received aggressive thoracic therapy for the primary tumor. Kudelin et al. describe a median OS of 26 months in patients with synchronous brain metastases who received surgical resection of the primary tumor and whole brain radiation therapy (WBRT). Due to the side effects of WBRT, it is usually no longer employed for singular or few brain metastases [12]. It has also been shown that patients with symptomatic NSCLC brain metastases have a significantly worse overall survival compared with asymptomatic brain metastases (7 months vs. 11 months) [13]. As of December 2021, two of our patients are still alive and have an OS and PFS of 28 and 35 months, respectively. One patient showed progressive disease 12 months after initial diagnosis, and passed away 31 months after initial diagnosis. This is of clinical importance because there are ongoing trials which evaluate immunochemotherapy in a neoadjuvant setting for lower stage NSCLC [14], and also trials that investigate the role of ICIs in NSCLC brain metastases [15].

NSCLC patients with synchronous, symptomatic brain metastases suffer from a severe, advanced stage of disease. The patients described in our case series showed a locally resectable primariy tumor with distant metastasis to the brain. So far, no studies concerning the combination of neoadjuvant immunochemotherapy with surgical resection and/or stereotactic radiosurgery of the brain metastasis followed by radical resection of the primary lung tumor have been published. The feasibility and safety (and much less the long-term results) of this concept are unknown.

The NCT02259621 [16], NEOSTAR [17], and ChiCTR-OIC-17013726 trials [18] have demonstrated the efficacy and feasibility of neoadjuvant monotherapy with anti PD-1 and anti PD-L1 drugs. However, none of these trials included patients with brain metastases.

In the NCT05012254 trial, the effect of immunochemotherapy on patients with brain metastases from NSCLC is being studied. A number of trials have recently been initiated testing a combination of stereotactic radiosurgery and immunochemotherapy, such as NCT03526900, NCT04787185, NCT04964960, NCT02978404, NCT02696993, and NCT04768075, showing that the combination of stereotactic radiosurgery with neoadjuvant immunochemotherapy for brain metastases and the induced abscopal effect is an understudied topic (Table 2).

None of these studies consider the question of local therapy to the primary tumor. However, the combination and the right timing of neoadjuvant immunochemotherapy and resection is a frequently discussed topic [19,20].

The CHESS study (NCT03965468) is currently investigating a similar therapy algorithm as is presented here. In the CHESS study, patients with synchronous oligometastatic NSCLC (including, but not limited to, patients with brain metastases, with at least one extracranial metastasis suited to stereotactic body radiation therapy) are treated with neoadjuvant therapy (paclitaxel, carboplatin, and durvalumab), as well as stereotactic radiosurgery of all metastases. If there is no disease progression after 3 months, definitive local treatment (surgical resection of the primary lung tumor or definitive radiotherapy) is performed. Afterwards, adjuvant therapy is administered with durvalumab for 12 months or until disease progression. This approach is very similar to the regimen presented in our case series, with the study still actively recruiting participants as of February 2022.

One of our patients showed a complete response of the primary tumor after neoadjuvant immunochemotherapy. This effect has also been described in the lung cancer mutation consortium (LCMC-3) study trial [21], which evaluated the effects of atezolizumab as a neoadjuvant and adjuvant therapy in patients with resectable UICC stage IB to IIIB disease. In the resected tissue from patient 1, extensive scarring with fibrotic and myxoid tissue remodeling was seen, as well as an accompanying lymphocytic infiltrate with evidence of giant cells and calcifications. In our series, this was also the patient with the highest PD-L1 expression (90%), which could be an indication that this patient collective could particularly benefit from neoadjuvant immunotherapy.

The administration of checkpoint inhibitors is almost always associated with inflammatory side effects due to the activation of the immune system. A study on 20 patients with resectable, non-metastasized NSCLC who received neoadjuvant treatment with two cycles of nivolumab described adhesions in either the interlobar fissure or surrounding the hilar and mediastinal nodal stations. This was also found to be the case in all three patients who had received immunochemotherapy with pembrolizumab. Although, in this study, the preoperative administration of ICIs was deemed to be safe and feasible, it is notable that 54% of procedures that had been attempted thoracoscopically or via robot-assisted surgery required conversion to open thoracotomy [22].

Resectable neoplasia, even at an advanced stage, can benefit from less invasive surgical approaches, such as video-assisted thoracoscopic surgery or robot-assisted surgery [23]. The possibility to dissect lymph nodes in the neck region via robot-assisted surgery has been demonstrated for other cancers, such as oropharyngeal squamous cell carcinoma [24]. However, when treating lung cancer, an advantage of an open surgical approach via posterolateral thoracotomy is the greater access for a systematic mediastinal lymph node dissection. According to the International Association for the Study of Lung Cancer (IASLC) 2009 thoracic lymph node mapping [25], the right hilar lymph nodes are situated in front and on top of the right main bronchus. Since patient 3 had a PET-positive lymph node in this region, the lymph node could be reached and completely resected after a wide surgical dissection of this area. Histopathological examination confirmed that the tumor had spread to the N3 lymph node in position 10R. Lymphonodal status should have been evaluated preoperatively via bronchoscopy with endobronchial ultrasound and transbronchial needle aspiration biopsy. However, it was possible to completely resect the lymph node metastasis, and until today, there has been no local recurrence in this region. So far, none of the three patients have developed an intrathoracic tumor recurrence, thereby avoiding local complications, such as recurrent nerve involvement [26,27], dyspnea, infection, or hemoptysis. In our opinion, this also speaks in favor of a surgical approach.

A combined therapy of ICIs and SRS appears beneficial [28], but is associated with an increase in pseudoprogression [29,30,31]. Also, ICI therapy of brain metastases may result in a greater occurrence of status epilepticus [32]. Due to the existing high tumor load, our patients could have been in danger of an increased occurrence of pseudoprogression and status epilepticus. However, only one in three patients had a seizure associated with the disease, which was successfully treated with levetiracetam.

When deciding on a course of therapy, the patient’s individual prognostic parameters should be considered. A meta-analysis based on retrospective studies from the pre-checkpoint era in oligometastatic NSCLC patients concluded that positive predictive factors for survival were aggressive therapy to the primary tumor, female gender, adenocarcinoma histology, as well as low (y)pT stage, and the absence of nodal disease [33]. Whether these factors remain the same when applying immunochemotherapy is yet unclear. Also, the patient’s general condition, medical history, social environment, and, not least, their willingness to embark on such a time-consuming and strenuous treatment plan need to be taken into account.

## 5. Conclusions

Our case series illustrates the feasibility of neurosurgery and/or radiosurgery followed by neoadjuvant immunochemotherapy and subsequent resection of the primary tumor in NSCLC patients with synchronous brain metastasis, with a very favorable outcome in two out of three patients. Though it is difficult to draw conclusions from such a small number of cases, prospective trials appear warranted.

## Figures and Tables

**Table 1:** Overview of the three patients.

	Patient 1	Patient 2	Patient 3
Age	56	53	58
Sex	m	m	f
Nicotine consumption	120 PYs	60 PYs	60 PYs
Disease	NSCLC adenocarcinoma	NSCLC adenocarcinoma	NSCLC adenocarcinoma
Pathology	TTF-1 positive, EGFR wt, ALK negative, BRAF wt, ROS-1 negative, K-RAS wt, PIK3CA positive, TP53 positive, HER2 wt, PD-L1: TPS 90% (high)	TTF-1 negative, EGFR wt, ALK negative, BRAF wt, ROS-1 negative, K-RAS mutation exon 2: codon 12, PD-L1: TPS 20% (moderate)	TTF-1 positive, EGFR wt, ALK negative, BRAF: not enough tissue for analysis, ROS-1 negative, K-RAS exon 2 mutation in codon 12, PD-L1: TPS 2% (moderate)
Clinical tumor stage	cT2 cN0 pM1b (BRA), UICC IVA	cT2b cN2 pM1b (BRA), UICC IVA	cT2 cN3 cM1c (BRA), UICC IVB
Location of primary tumor	Right lower pulmonary lobe	Right upper pulmonary lobe	Left lower pulmonary lobe
Initial neurological findings	Mnestic deficits, mild motoric aphasia (word-finding difficulty), and progressive numbness of the left side of the face	Oculomotor nerve deficits with ptosis, mydriasis, and abduction of the right eyeball	Impairment of fine motor skills, dizziness, apraxia, and weakness in the right arm
Location of BM	Right frontal lobe	Right temporal lobe	Left pre-central cortex and gyrus postcentralis
Karnofsky performance index before therapy	90%	90%	90%
Treatment of BM	Neurosurgery and SRS	Neurosurgery and SRS	SRS
BM radiation dose	21 Gy	Synchronous BM: 21 Gy, metachronous BM: 20 Gy	19 Gy
Neoadjuvant therapy	Cisplatin (75 mg/m^2^), pemetrexed (500 mg/m^2^), and pembrolizumab (200 mg)	Cisplatin (75 mg/m^2^), pemetrexed (500 mg/m^2^), and pembrolizumab (200 mg) (+2 cycles of pembrolizumab as maintenance therapy)	Cisplatin (75 mg/m^2^), pemetrexed (500 mg/m^2^), and pembrolizumab (200 mg)
Steroid application	Dexamethasone 4 mg/d for 3 months during neoadjuvant therapy	Dexamethasone 4 mg/d for 3 weeks after SRS	Dexamethasone 8 mg/d for 3 weeks before neoadjuvant therapy
Pulmonary function testing before pulmonary resection	FVC: 3.51 L (58%) FEV1: 2.36 L (51%) Tiffeneau index: 88%	FVC: 4.33 L (87%) FEV1: 2.46 L (65%) Tiffeneau index: 57%	FVC: 2.32 L (86%) FEV1: 1.49 L (68%) Tiffeneau index: 64%
Pulmonary resection	Posterolateral thoracotomy, subsegment S1-resection, right lower lobe resection, systematic lymph node dissection	Posterolateral thoracotomy, extrapleural extended upper lobectomy, wedge resections (S1, S5, S6), systematic lymph node dissection	Posterolateral thoracotomy, extended left lower lobe resection, wedge resection S1, systematic lymph node dissection
Tumor stage after lung resection	ypT0 ypN0 (0/42), L0, V0, R0, Gx, pM1b (BRA), UICC IVA	ypT2b ypN0 (0/22), L0, V0, Pn0, R0, Gx, pM1b (BRA), UICC IVA	ypT2a ypN3 (5/23), L1, V1, Pn0, R0, cM1c (BRA), UICC IVB
Maintenance therapy	200 mg per cycle (ongoing)	200 mg per cycle (discontinued after 2 cycles because of progressive disease)	200 mg per cycle (finished after 24 months)
Current Karnofsky performance index	100%	0%	100%

Abbreviations: SRS: stereotactic radiosurgery, BM: brain metastasis.

**Table 2:** Overview of the current clinical trial landscape for NSCLC patients with brain metastases.

Trial	Institution	Therapy	Inclusion Criteria	Tumor Histology	Local Treatment of Primary Tumor	Local Treatment of BM	No. of Patients	Study Start Date	Recruiting
NCT05012254 (NIVIPI-Brain)	Spanish Lung Cancer Group	Nivolumab plus Ipilimumab and 2 cycles of platinum-based chemotherapy	Synchronous and metachronous BM—6 months after other chemotherapy treatment has finished, asymptomatic, or oligosymptomatic BM	NSCLC	-	BM must not be suitable for resection or focal RT	71 planned	November 2021	yes
NCT03526900 (ATEZO-BRAIN)	Spanish Lung Cancer Group	Atezolizumab and platinum-based chemotherapy	Synchronous and metachronous asymptomatic BM	Nonsquamous NSCLC	-	WBRT or SRS only in case of brain progression	43	July 2018	active, not recruiting
NCT04787185 (STRAIT-LUC)	Azienda Ospedaliero-Universitaria Careggi	Immunotherapy	Up to 10 BMs treatable with RS or HFSRT	NSCLC	-	RS or HFSRT	50 planned	April 2020	yes
NCT04964960	University of Kentucky	Pembrolizumab and platinum-based chemotherapy	Asymptomatic BM (less than 10 lesions)	NSCLC	-	-	45 planned	December 2021	yes
NCT02978404	Centre hospitalier de l’Université de Montréal	Nivolumab	BMs with a combined maximum disease volume of 10cc	NSCLC, SCLC, melanoma, or ccRCC	-	SRS during Nivolumab treatment	26	June 2017	active, not recruiting
NCT02696993	MD Anderson Cancer Center	Nivolumab (plus Ipilimumab)	At least one BM >= 0.3 cm amenable to radiation therapy	NSCLC	-	SRS or WBRT	88 planned	December 2016	yes
NCT04768075	Guangdong Association of Clinical Trials	Camrelizumab and platinum-based chemotherapy	Symptomatic and asymptomatic BM	NSCLC	-	SRT or WBRT	200 planned	March 2021	not yet
NCT03965468 (CHESS)	European Thoracic Oncology Platform	Durvalumab and platinum-based chemotherapy	Up to 3 metastases including BM, must have at least 1 extracerebral metastasis	NSCLC	Resection or definitive radiotherapy	SBRT for extracranial metastasis, radiosurgery, or neurosurgery for BM	47 planned	November 2019	yes

Abbreviations: BM: brain metastasis, ccRCC: clear cell renal cell carcinoma, HFSRT: hypofractionated stereotactic radiation therapy, RS: radiosurgery, SBRT: stereotactic body radiation therapy, SCLC: small cell lung cancer, SRS: stereotactic radiosurgery, SRT: stereotactic radiation therapy, WBRT: whole brain radiation therapy.

## Data Availability

The original contributions presented in the study are included in the article material. Further inquiries can be directed to the corresponding author.
